# How does genetic architecture affect eco-evolutionary dynamics? A theoretical perspective

**DOI:** 10.1098/rstb.2020.0504

**Published:** 2022-07-18

**Authors:** Masato Yamamichi

**Affiliations:** ^1^ School of Biological Sciences, The University of Queensland, St. Lucia, Brisbane, QLD 4072, Australia; ^2^ Department of International Health and Medical Anthropology, Institute of Tropical Medicine, Nagasaki University, Nagasaki 852-8523, Japan

**Keywords:** allele dominance, epistasis, linkage disequilibrium, number of loci, phenotypic plasticity, rapid evolution

## Abstract

Recent studies have revealed the importance of feedbacks between contemporary rapid evolution (i.e. evolution that occurs through changes in allele frequencies) and ecological dynamics. Despite its inherent interdisciplinary nature, however, studies on eco-evolutionary feedbacks have been mostly ecological and tended to focus on adaptation at the phenotypic level without considering the genetic architecture of evolutionary processes. In empirical studies, researchers have often compared ecological dynamics when the focal species under selection has a single genotype with dynamics when it has multiple genotypes. In theoretical studies, common approaches are models of quantitative traits where mean trait values change adaptively along the fitness gradient and Mendelian traits with two alleles at a single locus. On the other hand, it is well known that genetic architecture can affect short-term evolutionary dynamics in population genetics. Indeed, recent theoretical studies have demonstrated that genetic architecture (e.g. the number of loci, linkage disequilibrium and ploidy) matters in eco-evolutionary dynamics (e.g. evolutionary rescue where rapid evolution prevents extinction and population cycles driven by (co)evolution). I propose that theoretical approaches will promote the synthesis of functional genomics and eco-evolutionary dynamics through models that combine population genetics and ecology as well as nonlinear time-series analyses using emerging big data.

This article is part of the theme issue ‘Genetic basis of adaptation and speciation: from loci to causative mutations’.

## 1. Introduction

The traditional assumption in ecology and evolutionary biology has been that evolutionary processes are much slower than contemporary ecological processes [1,2]. Recent studies, on the other hand, have demonstrated that rapid adaptive evolution (i.e. allele frequency changes in populations over just a few generations) is common and can be rapid enough to affect ongoing ecological processes including population, community and even ecosystem dynamics [3–9]. Selection pressure is often fluctuating [10] and temporally fluctuating selection can make evolution rapid over short time scales and can cancel out the evolutionary responses across longer time scales [5,8]. Because ecological processes alter fitness landscapes and drive adaptive evolution [11], there should be an interplay between ecological and evolutionary processes. The resultant feedback between ecological processes and rapid adaptive evolution is called eco-evolutionary dynamics [12]. Eco-evolutionary dynamics is one of the most active research areas in ecology and evolutionary biology [13–20] not only for the synthesis of these two basic sciences, but also for conservation and management of wild organisms rapidly evolving in response to drastic environmental changes [21–23].

**Figure 1.**
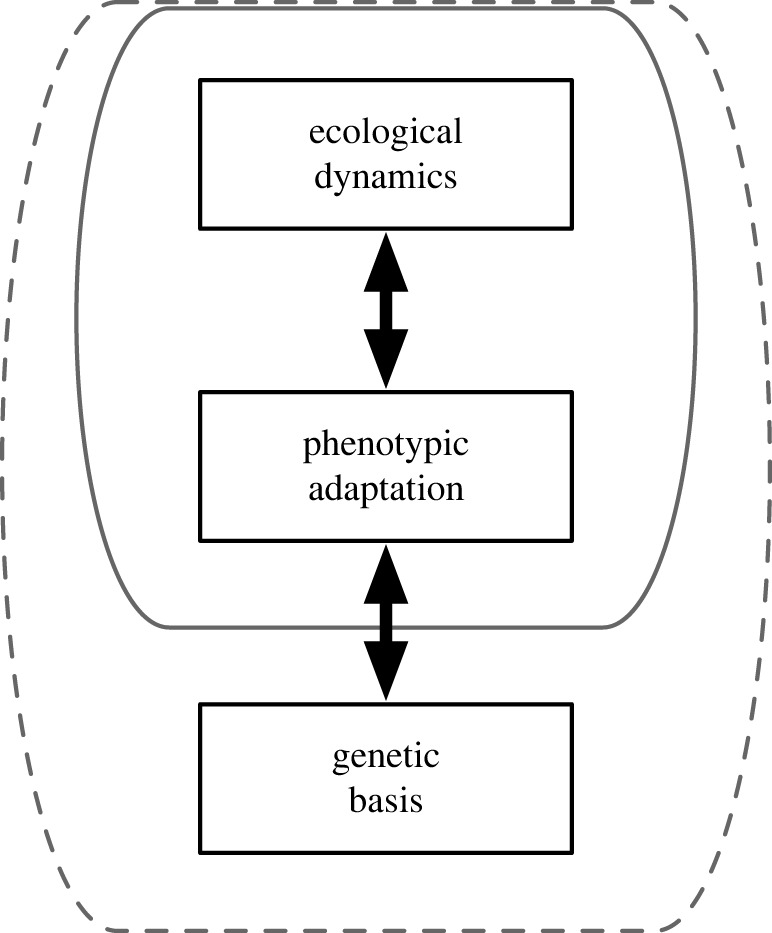
The conceptual framework of eco-evolutionary feedbacks (after [26]). Previous studies in eco-evolutionary dynamics tended to focus on feedbacks between ecological processes and phenotypic adaptation (indicated by the solid line). Including genetic basis of phenotypic adaptation (as indicated by the dashed line) may improve our understanding of eco-evolutionary dynamics. Note that the figure seems to be suggesting that the three components are separate, but they are confounded with one another.

Although studies of eco-evolutionary dynamics combine insights from ecology and evolutionary biology and are inherently interdisciplinary, it seems that research on eco-evolutionary feedbacks has been mostly conducted from the perspective of ecology. For example, the finding of evolutionary cycles where prey defence evolution changes the phase lag between predator and prey densities from a quarter-period to a half-period [24,25] was surprising for ecologists, but dynamics of prey defence traits there might not be so novel for evolutionary biologists. According to the Web of Science, about 58% of papers in a search of ‘eco-evo* dynamics’ were categorized as ‘Ecology’ whereas 30% were ‘Evolutionary Biology’ and 11% of papers were ‘Genetics Heredity’ (searched on 2 March 2022). Researchers have tended to focus on feedbacks between ecological dynamics and adaptation at the phenotypic level (the solid line surrounding these categories in figure 1) instead of including the genetic architecture (see Glossary) of evolutionary processes (the dashed line in figure 1) (see the following section). Treating genetic details as a black box (the ‘phenotypic gambit’ in evolutionary ecology [27]) is a powerful, simplifying and convincing approach for understanding complex long-term evolutionary dynamics. However, short-term evolutionary dynamics may be more constrained by the genetic architecture of phenotypic adaptation, especially because many such short-term adaptative changes are driven by a limited amount of standing genetic variation instead of a tremendous amount of de novo mutations [28]. Thus, understanding genetic architecture will be important for deepening our understanding of eco-evolutionary dynamics.

A similar argument about the potential importance of mechanisms of adaptation has been made for the difference between rapid evolution and phenotypic plasticity. Both rapid evolution and phenotypic plasticity are trait changes that often increase an individual's fitness and are rapid enough to affect ecological dynamics [5,29,30]. It may be difficult for us to differentiate them when we observe adaptive trait changes in the wild (but see [31]), although plasticity is not necessarily adaptive. Some studies suggested that genetic evolution, phenotypic plasticity and even behavioural changes based on learning processes can be described by a quantitative trait model (see the following section) simply through changing the speed of trait adaptation (e.g. [32–34]). However, theoretical studies have proposed that phenotypic plasticity may be better at stabilizing population cycles due to faster responses to environmental changes [35–37] and may not cause antiphase predator–prey cycles unlike rapid evolution because plastic changes are not directly affected by the local fitness gradient [37,38]. Indeed, experimental studies on the rapid evolution of prey defence traits in zooplankton–phytoplankton microcosm systems showed antiphase cycles [24,39], whereas those on inducible defence did not find antiphase cycles [40].

While some studies have pointed out the potential importance of genomic studies in eco-evolutionary dynamics [41–44], the dynamic consequences of genetic architectures on eco-evolutionary dynamics have to date not been well recognized. Here I review theoretical results on the effects of genetic architecture on evolutionary and eco-evolutionary dynamics and propose a future direction where genetic and genomic studies deepen our understanding of eco-evolutionary dynamics by combining dynamic models and nonlinear time-series analyses.

## 2. Common approaches in eco-evolutionary dynamics

For understanding the effects of rapid evolution on ecological dynamics, empirical researchers often compared ecological dynamics when the focal species under selection has only a single allele at the focal locus versus dynamics with multiple alleles and so can evolve (or, in the case of asexually reproducing species, dynamics with a single clonal genotype versus dynamics with multiple clonal genotypes; e.g. [24,45–47]). Even with a single genotype of asexual organisms, de novo mutations may produce genetic variation and eventually cause rapid evolution [39,48]. However, as long as experimental periods are short, mutation rates are small and generation time is not relatively short, it will be possible to observe ecological dynamics without rapid evolution [49].

It should be noted that there are three types of empirical studies: (1) studies examining the effects of ongoing rapid evolution on ecological dynamics (e.g. [24,39]), (2) studies examining the effects of evolved traits (usually after short evolution experiments) on ecological dynamics (e.g. [50–52]) and (3) studies examining the effects of genetic variation (without evolutionary changes) on ecological dynamics in short-term experiments (e.g. [45]). Case (1) may be further divided into (1a) continuous eco-evolutionary dynamics where genetic variation is maintained by selection (e.g. [24]) and (1b) transient eco-evolutionary dynamics where selection eventually removes genetic variation (e.g. [53]). Although genetic variation is a prerequisite of rapid evolution in most situations, rapid evolution does not always occur during the experiments in cases (2) and (3). Studies in ‘community genetics’ tend to use plant traits, and thus to consider cases (2) and (3) [54], while theoretical studies often consider case (1). Genetic architectures may become important in empirical studies of the case (1) type.

In theoretical studies, common approaches assume continuous quantitative traits controlled by many loci with small effects:

2.1 $$\begin{array}{rl} \frac{d\bar{z}}{dt}=\; & f(\bar{z},\;N)\\ \mathrm{and}\;\frac{dN}{dt}=\; & g(\bar{z},\;N), \end{array}\}$$

where $\bar{z}$ is a mean value of a quantitative trait, *N* is a population density, and *f* and *g* represent their dynamics [12,18,20]. Mean trait dynamics is often represented by

2.2 $$\frac{d\bar{z}}{dt}=\nu \frac{\partial \bar{W}}{\partial \bar{z}},$$

where *ν* is additive genetic variance and $\bar{W}$ is population mean fitness (i.e. the *per capita* growth rate: d*N*/*N*d*t*) [32,55]. Here the mean trait changes along the local fitness gradient to increase the fitness (e.g. [33,34,38,56–59]).

Some studies employed models of discrete Mendelian traits with two alleles in a single locus (e.g. [60]) or a clonal model,

2.3 $$\begin{array}{rl} \frac{dN_{1}}{dt}=\; & f_{1}(N_{1},\;N_{2})\\ \mathrm{and}\;\frac{dN_{2}}{dt}=\; & f_{2}(N_{1},\;N_{2}), \end{array}\}$$

where *N_i_* represents the density of a clone (genotype) *i* in an asexual organism such as bacteria and algae (e.g. [25,35,36,39,48]). This can be re-written as

**Figure 2.**
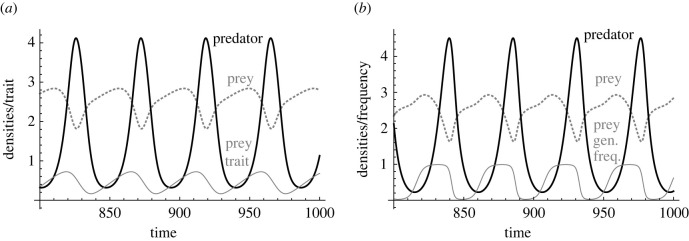
Antiphase predator–prey cycles in (*a*) a quantitative trait model [56] and (*b*) a clonal model [25]. Black solid lines and grey dotted lines represent predator and prey densities, respectively. Grey solid lines are (*a*) prey trait and (*b*) prey genotype frequency, respectively, and higher values indicate less defended states.

2.4 $$\begin{array}{rl} \frac{dp}{dt}=\; & p(1-p)\left(\frac{1}{N_{1}}\frac{dN_{1}}{dt}-\frac{1}{N_{2}}\frac{dN_{2}}{dt}\right)\\ \mathrm{and}\;\frac{dN_{T}}{dt}=\; & f_{1}(N_{1},\;N_{2})+f_{2}(N_{1},\;N_{2}), \end{array}\}$$

where *N_T_* = *N*_1_ + *N*_2_ and *p* = *N*_1_/*N_T_*. Note that equation (2.4) corresponds to equation (2.1): *p*(1 − *p*) is the additive genetic variance and the difference between the *per capita* growth rates represents the fitness gradient. While the additive genetic variance *ν* is often assumed to be a fixed parameter in equation (2.2), the variance *p*(1 − *p*) changes depending on the clonal frequency *p* in equation (2.4). Despite the difference, the two approaches can produce very similar dynamics [57,61]. For example, predator–prey antiphase cycles with quantitative traits [56], and those with two clonal genotypes [25,48] are basically very similar (figure 2). Theoreticians have sometimes used an Adaptive Dynamics approach (i.e. evolutionary invasion analysis) assuming asexual reproduction as well (e.g. [62]), but a common assumption seems to be that genetic architectures do not matter and can be safely ignored for understanding eco-evolutionary dynamics [63].

## 3. Effects of genetic architecture on evolutionary dynamics

**Figure 3.**
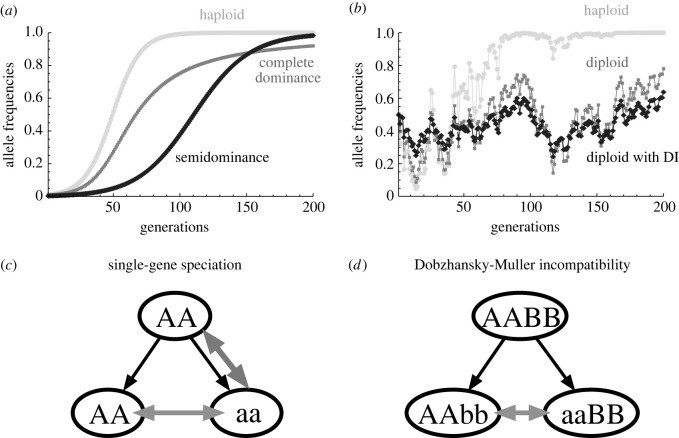
Examples of the effects of genetic architectures on evolutionary dynamics. (*a*) The effects of ploidy and allele dominance on evolutionary dynamics under directional selection. Haploid (light grey), diploid with complete dominance (grey) and diploid with semidominance (black) are shown. (*b*) The effects of ploidy and delayed inheritance (DI) on evolutionary dynamics under temporally fluctuating selection [78]. Haploid (light grey), diploid with complete dominance (grey) and diploid with DI (black) are shown. (*c,d*) The effects of the number of loci on speciation processes. (*c*) Single-gene speciation from an ancestral population with an allele A to two populations with alleles A and a where there is reproductive incompatibility between alleles A and a (shown by grey arrows). Because of the incompatibility, it is difficult for a mutant allele a to increase in an ancestral population with a resident allele A. (*d*) Speciation from an ancestral population with alleles A and B to two populations with alleles A, B, a and b where there is Dobzhansky–Muller incompatibility between alleles a and b due to epistasis. Mutant alleles a and b can increase in an ancestral population without incompatibility unlike the model of single-gene speciation.

In evolutionary biology, especially in population genetics, it is well known that genetic architecture can affect evolutionary dynamics. Genetic architectures themselves can evolve in response to selection over long time scales (e.g. [64]), but short-term evolution is constrained by the relationships between genotypes and phenotypes. Previous studies demonstrated that a single gene can have large phenotypic consequences in insects [65,66], mollusks [67], fish [68,69], mammals [70–72] and plants [73,74]. Although there is likely to be publication bias and many adaptive traits are likely quantitative with many loci that have small effects [75,76], it is meaningful to start from models with one locus or two loci for heuristic purposes [77]. Here I outline three examples: the effects of ploidy and allele dominance on the speed of allele fixation (figure 3*a*), the effects of ploidy and maternal effects on the maintenance of genetic variation under temporally fluctuating selection (figure 3*b*), and the required number of loci in speciation (figure 3*c*,*d*).

Probably the simplest example is evolutionary dynamics under directional selection (figure 3*a*). Haploid inheritance is the most sensitive to selection, whereas complete dominance in diploid inheritance can delay evolutionary responses to selection due to a mismatch between genotypes and phenotypes: heterozygotes include a recessive allele but have a dominant phenotype. When dominant mutant alleles are selected for, they can quickly increase when rare, but it is difficult for them to remove the resident recessive alleles unlike semidominance. With genetic drift in finite populations, frequency dynamics when alleles are rare are important for fixation, and thus adaptive alleles are more likely to be dominant (i.e. Haldane's sieve: [79]).

With temporally fluctuating selection [10], haploid inheritance is so sensitive to selection pressure that it cannot maintain genetic variation: an allele with the highest geometric mean fitness dominates and other alleles will be lost from a population (figure 3*b*) [80]. On the other hand, the maintenance of genetic variation is possible in diploid inheritance with complete dominance because alleles can be stored in heterozygotes when they are not favoured [81,82]. This is what we call the storage effect [83]. These days, researchers tend to think that overlapping generations play a primary role for buffered population growth of the storage effect [83,84], but genetic architecture can also work for buffering. As like complete dominance, a maternal genetic effect where maternal genotypes determine offspring phenotypes (delayed inheritance (DI)) further blurs the relationship between genotypes and phenotypes and makes the maintenance of genetic variation easier [78]. Note that there are a few other mechanisms that have been demonstrated to maintain genetic diversity (e.g. reversal of dominance) and they are summarized in Bertram and Masel [85].

When an ancestral population splits into two populations, researchers have suggested speciation is unlikely when reproductive incompatibility is caused only by a single locus with two alleles. This is because there is reproductive incompatibility between alleles in this single-gene speciation scenario and hence it is difficult for a mutant allele to increase when rare (figure 3*c*) [86,87]. When there are two loci with epistasis, on the other hand, speciation can occur without difficulty: this is called Dobzhansky–Muller reproductive incompatibility (figure 3*d*) [88–90]. In this case, reproductive incompatibility occurs between mutant alleles at the two loci due to epistasis. Thus, the number of loci affecting reproductive incompatibility determines the outcome of the speciation processes.

## 4. Effects of genetic architecture on eco-evolutionary dynamics

As shown in the previous section, genetic architectures can affect evolutionary dynamics and thus eco-evolutionary dynamics as well. Here I introduce recent theoretical studies that showed the potential effects of the genetic architecture on eco-evolutionary dynamics. In future empirical studies, it may become possible to compare eco-evolutionary dynamics with different genetic architecture (e.g. dynamics with haploid inheritance versus dynamics with diploid inheritance) directly based on the following theoretical predictions as like studies on rapid evolution and phenotypic plasticity. There are many possible combinations of ecological dynamics (e.g. population extinction and population cycles) and genetic details (e.g. the number of loci and recombination), and there are a few studies that have explored some of them (table 1).

**Table 1.** Theoretical studies that combine ecological dynamics and genetic structure. Note that sexual reproduction, recombination and ploidy are fundamentally tightly related.

		ecological dynamics	
		population extinction (evolutionary rescue)	predator–prey cycles (including apparent and exploitative competition)
genetic structure	number of loci	Orr & Unckless [91], Gomulkiewicz *et al.* [92], Kardos & Luikart [93]	Yamamichi & Ellner [94]
	recombination/epistasis	Schiffers *et al.* [95], Uecker & Hermisson [96]	Patel & Bürger [97]
	clonal versus sexual reproduction/ploidy	Orive *et al.* [98], Uecker [99], Peniston *et al.* [100]	Schreiber *et al.* [60], Doebeli & Koella [101], Doebeli [102], Bolnick *et al.* [103]

Evolutionary rescue is probably the most interdisciplinary topic in eco-evolutionary dynamics, with work from ecologists, evolutionary biologists, population geneticists and medical researchers [104–106]. Evolutionary rescue is a phenomenon where rapid adaptive evolution prevents population extinction in the face of an environmental change [107]. It is not only important for conservation and wildlife management, but also for medicine where researchers seek to prevent evolutionary rescue of bacteria from suppression by antibiotics [104]. Gomulkiewicz & Holt [107] originally examined a quantitative-genetic model (as like equation (2.2)) and a one-locus model (as like equation (2.3)) and obtained qualitatively similar results. Orr & Unckless [91] showed that it is difficult for a single locus to adapt to rapid environmental change compared with the case for multiple loci where any one of them can rescue the population. On the other hand, Gomulkiewicz *et al*. [92] showed that increasing the number of loci can decrease the speed of adaptation and prevent the resultant rescue from extinction because selection per locus is weakened. More recently, Kardos & Luikart [93] demonstrated that population extinction is less likely in models with polygenic architectures compared with models with large-effect loci due to higher short-term evolutionary potential. Uecker & Hermisson [96] analysed a model where evolutionary rescue depends on mutations at two loci and found complex effects of recombination on extinction because recombination generates and breaks up favourable gene combinations. These studies suggest that models at the extremes of either a single locus or infinitely many loci behave similarly, whereas models with intermediate numbers of loci may show complex dynamics.

**Figure 4.**
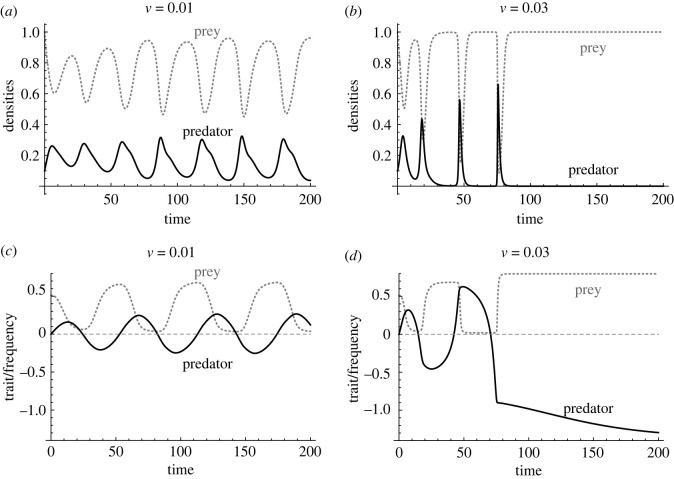
Rapid predator evolution can result in predator extinction in genetically asymmetric coevolution (coevolution between a prey's Mendelian trait and a predator's quantitative trait) [94]. Predation is more successful when traits match (i.e. a bidirectional axis of vulnerability [108]) due to, for example, handedness of snails and snakes. (*a*,*c*) Persistent predator–prey population cycles (*a*) and trait coevolution (*c*) when the additive genetic variance of the quantitative trait, *v* = 0.01. (*b*,*d*) Predator extinction (*b*) due to large amplitudes in trait coevolution (*d*) when additive genetic variance of the quantitative trait, *v* = 0.03. Black solid lines and grey dotted lines represent predator and prey species, respectively.

Predator–prey population dynamics has been a central topic in eco-evolutionary dynamics since the seminal experimental papers on antiphase and cryptic cycles driven by rapid evolution [24,48]. Because those studies considered defence evolution of asexually reproducing algae, genetic details have not been considered intensively [48,57]. Yamamichi & Ellner [94] modelled antagonistic coevolution between the Mendelian trait of a prey and the quantitative trait of its predator inspired by a snake-snail predator–prey system [67]. They found that rapid predator evolution can result in predator extinction (figure 4*a*,*b*) unlike coevolution between Mendelian traits or between quantitative traits. This is because evolution of the prey's discrete trait can throw off tracking by the predator's continuous trait as the amplitudes of coevolutionary cycles amplify, especially with complete allele dominance (figure 4*c*,*d*). On the other hand, Schreiber *et al*. [60] examined the effects of ploidy (haploid versus diploid) on species coexistence and showed that diploid inheritance can stabilize community dynamics with exploitative and apparent competition due to the inefficacy of selection. More recently, Patel & Bürger [97] explored how recombination in predator species affects apparent competition of two prey species and found a novel feedback between predator density, total prey density and linkage disequilibrium in the predator induced by epistatic fitness effects of linked loci.

While previous studies of eco-evolutionary dynamics have tended to focus on evolutionary rescue and predator–prey interactions, it will be interesting to examine other ecological dynamics such as competitive and mutualistic interactions [109] as well as host–parasite dynamics [110]. In addition, speciation processes can be studied from the perspective of eco-evolutionary dynamics. For example, rapid evolution in reproductive character displacement (reinforcement) can prevent population extinction by weakening reproductive interference and positive frequency-dependence in community dynamics due to incomplete reproductive isolation [111,112]. It may be interesting to study how the genetic basis of speciation (speciation genes: figure 3*c*,*d*) affects eco-evolutionary dynamics.

## 5. Conclusion and future directions

Previous studies have shown that genetic details can affect evolutionary and eco-evolutionary dynamics (figures 3 and 4). However, few theoretical studies have examined the effects of genetic architectures on eco-evolutionary dynamics (table 1). Therefore, more studies are needed of eco-evolutionary dynamics that integrate genetics, evolutionary biology and ecology (figure 1). Recent studies have emphasized the importance of the analogy between community ecology and population genetics [113,114], but the integrated eco-evolutionary framework (figure 1) will be another important step for population biology synthesizing population genetics and population ecology.

There are many ways to add genetic details to simple eco-evolutionary models such as equations (2.1)–(2.4), including epigenetics, pleiotropy and allele dominance in addition to the number of loci, varying phenotypic effects of loci, recombination, epistasis, ploidy and sexual versus asexual reproduction (table 1). Indeed some researchers made the quantitative trait model (equation (2.2)) more realistic by considering trait variance dynamics [115], bimodal trait distributions [116] and evolutionary diversification [117]. However, complex models are not always better than simple ones. All models are wrong, and hence it is important to ask when we need to care about genetic bases of ecologically important traits. Indeed, the quantitative trait and clonal models show similar eco-evolutionary dynamics (figure 2), and models with 20, 100 or 1000 loci may show very similar dynamics [92]. In this case, simply estimating additive genetic variance of fitness-associated traits may be better than considering genetic basis. Accumulating more theoretical studies should reveal conditions where the details can be safely ignored.

Because of rapid developments of molecular biological techniques, it is now possible to investigate genetic basis of ecologically important traits in non-model organisms [118,119]. This ecological and evolutionary functional genomics will not only promote our understanding of past evolutionary processes, but also contribute to studies on eco-evolutionary dynamics [41–44]. Some organisms are often used for genomic studies as well as studies on eco-evolutionary dynamics. Thus, it will be possible to connect genome structure and eco-evolutionary dynamics by using, for example, baker's yeast (*Saccharomyces*: [53]), green algae (*Chlamydomonas*: [120]), waterflea (*Daphnia*: [121]), threespine sticklebacks (*Gasterosteus*: [46]), thale cress (*Arabidopsis*: [122]) and poplars (*Populus*: [54]). Even with the genomic resources, however, genomics of eco-evolutionary dynamics is still in its infancy due to its inherent difficulty. How can we understand the relationship between fitness and traits in addition to the relationship between traits and genomes? When selective landscapes vary through time, when should the architecture be studied? If the architecture varies over time, what can be learned? Indeed, previous studies found that various genetic bases can exist behind the same evolutionary responses [119,120,123]. This may be a part of the reason why there are not so many empirical studies on genomics of eco-evolutionary dynamics despite the previous perspective papers [41–44].

Lastly, I propose three possible research directions that would combine genomic data and eco-evolutionary dynamics with a guide of theoretical modelling: backward inferences based on genomic data, nonlinear time-series data analyses and genome-wide association studies. First, if we know how eco-evolutionary dynamics affect genomic patterns (e.g. how evolutionary rescue affects selective sweep and genetic hitchhiking of linked neutral alleles: [124]) by using population genetic models, then it may even be possible for us to detect past eco-evolutionary dynamics from population genomic data. This may be an interesting approach for transient dynamics such as evolutionary rescue [124] as well as continuous dynamics such as coevolutionary cycles [125]. Second, when time series of genomic data are available (e.g. [126,127]), nonlinear time-series data analyses such as empirical dynamic modelling (EDM) [128] and transfer entropy [129] may make it possible to infer causal relationships between time-series data of allele frequencies in single-nucleotide polymorphisms (SNPs), expression patterns, fitness and population densities. Currently, it is very difficult to obtain such a huge amount of time-series data, but it may become possible to collect data of wild organism more easily in the near future through automated monitoring with advanced techniques such as environmental DNA [130], machine learning for camera trap data [131], mobile DNA sequencers and unmanned aerial vehicles [132]. Then, we may be able to use EDM to re-construct attractors from time-series data based on Takens’ theorem and to infer causal relationships between genomic data and ecological processes [128,133,134]. Based on the time-series analyses, we may be able to draw integrated networks of eco-evolutionary dynamics including gene interactions, trait interactions and species interactions [76,135]. Note that eco-evolutionary dynamics can be cryptic (i.e. eco-evolutionary dynamics may appear like purely ecological expectations) [48,136], and in this case, it may be difficult to infer causality solely from time-series analyses. In addition, because fitness is an emergent property of many traits, even when there are alleles of moderate effect on individual fitness-associated traits, their individual effect on resultant eco-evolutionary processes is likely to be quite small because of a polygenic basis [137]. This considerable hurdle in many empirical systems may be addressed by time-series analyses, if researchers can obtain big data from genome, epigenome, fitness, trait dynamics and ecological dynamics. Finally, even when the data are not especially rich, it will be interesting to examine associations between genetic markers (e.g. SNPs and structural variants) and key ecological parameters (e.g. population densities of the focal species or community compositions on the focal host species). This may be done by conducting genome-wide association studies that examine associations between genetic and epigenetic patterns with ecological dynamics (instead of phenotypic traits) [138] as well as differentiation outlier methods that screen for alleles that show large genetic differentiation between populations that exhibit different ecological patterns [139]. In this context, theoretical models will be useful for understanding the entangled interactions between genes, traits and species even in this era of big data.

## Data Availability

This article has no additional data.

## Glossary

Epistasis: the phenomenon where the effects of one gene on a phenotype is affected by the expression of other gene(s).
Genetic architecture: the genetic basis underlying a phenotypic trait.
Haldane's sieve: the bias against the fixation of recessive beneficial mutations.
Linkage disequilibrium: the nonrandom assortment of alleles at different loci (i.e. the deviation from independent association).
Pleiotropy: the phenomenon where one gene affects two (or more) phenotypic traits.
Ploidy: the number of complete sets of chromosomes in the nucleus of a cell.
